# Ethyl 1,6-dimethyl-2-oxo-4-(quinolin-4-yl)-1,2,3,4-tetra­hydro­pyrimidine-5-carboxyl­ate

**DOI:** 10.1107/S1600536810023482

**Published:** 2010-06-23

**Authors:** Roman I. Zubatyuk, Oleg V. Shishkin, Heiko Ihmels, Iryna A. Lebedyeva, Mykhaylo V. Povstyanoy

**Affiliations:** a STC "Institute for Single Crystals" NAS of Ukraine, 60 Lenina Ave., Kharkiv, Ukraine; b University of Siegen, FB 8, Adolf Reichwein Strasse 2, 57068 Siegen, Germany; c Department of Organic and Biochemical Synthesis, Kherson National Technical University, 24 Berislavske Highway, Kherson 73008, Ukraine

## Abstract

In the title compound, C_18_H_19_N_3_O_3_, the tetra­hydro­pyrimidone ring adopts a distorted boat conformation. In the crystal structure, inter­molecular N—H⋯O hydrogen bonds link the mol­ecules into centrosymmetric dimers, which are further linked *via* inter­molecular C—H⋯π inter­actions. In addition, an intra­molecular C—H⋯O hydrogen bond occurs.

## Related literature

It has been proposed that the combination of the biologically active dihydro­pyrimidine subunit with a DNA inter­calator (Waring, 2006 ▶; Hannon, 2007 ▶; Ihmels & Otto, 2005 ▶) may lead to a new class of DNA-targeting drugs (Neidle & Thurston, 2005 ▶; Braña *et al.*, 2001 ▶). Thus, a classical DNA intercalator, namely quinoline (Denny, 2003 ▶; Kharatishvili *et al.*, 1997 ▶; Aislabie *et al.*, 1990 ▶), was employed as quinoline-4-carbaldehyde in the Biginelli (1893 ▶) reaction that leads to the title compound. For the biological activity of pyrimidine-containing compounds, see: Goldmann & Stoltefuss (1991 ▶); McKinstry & Reading (1944 ▶); Kappe (2000 ▶); Luo *et al.* (2004 ▶). For van der Waals radii, see: Zefirov & Zorky (1989 ▶).
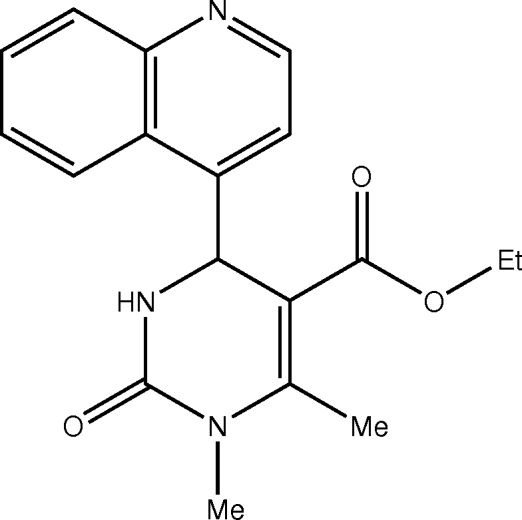


## Experimental

### 

#### Crystal data

C_18_H_19_N_3_O_3_
*M* *_r_* = 325.36
Triclinic, 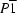

*a* = 8.5039 (10) Å
*b* = 9.4637 (11) Å
*c* = 10.7503 (11) Å
α = 110.799 (10)°
β = 95.807 (9)°
γ = 97.339 (9)°
*V* = 792.07 (17) Å^3^
*Z* = 2
Mo *K*α radiation
μ = 0.10 mm^−1^
*T* = 293 K
0.4 × 0.3 × 0.2 mm

#### Data collection

Oxford Diffraction Xcalibur diffractometer with Sapphire3 detector
Absorption correction: multi-scan (*CrysAlis PRO*; Oxford Diffraction, 2009 ▶) *T*_min_ = 0.975, *T*_max_ = 1
8517 measured reflections
4607 independent reflections
2810 reflections with *I* > 2σ(*I*)
*R*_int_ = 0.017

#### Refinement

*R*[*F* ^2^ > 2σ(*F* ^2^)] = 0.044
*wR*(*F* ^2^) = 0.117
*S* = 0.99
4607 reflections
220 parameters
H-atom parameters constrained
Δρ_max_ = 0.30 e Å^−3^
Δρ_min_ = −0.17 e Å^−3^

### 

Data collection: *CrysAlis PRO* (Oxford Diffraction, 2009 ▶); cell refinement: *CrysAlis PRO*; data reduction: *CrysAlis PRO*; program(s) used to solve structure: *Superflip* (Palatinus & Chapuis, 2007 ▶); program(s) used to refine structure: *SHELXL97* (Sheldrick, 2008 ▶); molecular graphics: *PLATON* (Spek, 2009 ▶); software used to prepare material for publication: *publCIF* (Westrip, 2010 ▶).

## Supplementary Material

Crystal structure: contains datablocks I. DOI: 10.1107/S1600536810023482/lx2153sup1.cif

Structure factors: contains datablocks I. DOI: 10.1107/S1600536810023482/lx2153Isup2.hkl

Additional supplementary materials:  crystallographic information; 3D view; checkCIF report

## Figures and Tables

**Table 1:** Hydrogen-bond geometry (Å, °) *Cg*1 is the centroid of the C8–C13 ring.

*D*—H⋯*A*	*D*—H	H⋯*A*	*D*⋯*A*	*D*—H⋯*A*
N2—H2⋯O3^i^	0.86	2.03	2.8701 (16)	164
C17—H17*A*⋯O2	0.96	2.16	2.8187 (19)	124
C16—H16*C*⋯*Cg*1^ii^	0.96	2.66	3.603 (2)	168

Symmetry codes: (i) 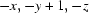; (ii) 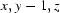.

**Fig. 1.**
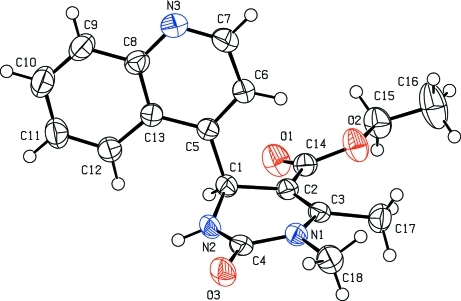
The molecular structure of the title compound with the atom numbering scheme. Displacement ellipsoids drawn at the 50% probability level. H atoms are presented as a small spheres of arbitrary radius.

**Fig. 2.**
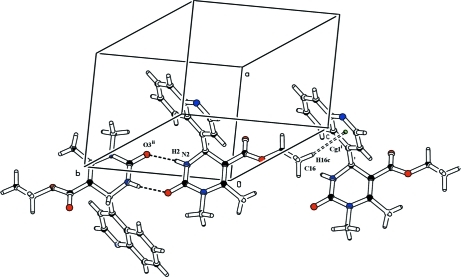
N—H···O and C—H···π interactions (dotted lines) in the crystal structure of the title compound. Cg denotes the ring centroid. [Symmetry codes: (i) x, - y + 1, z ; (ii) - x, - y + 1, - z. ]

## supplementary crystallographic information

### Comment

Pyrimidine-containing compounds exhibit a wide spectrum of biological activity (Goldmann & Stoltefuss, 1991). For example, dihydropyrimidines derivatives (DHPMs) may be applied as antimicrobial (McKinstry & Reading, 1944), anti-inflammatory (Kappe, 2000), anticarcenogenic drugs (Luo *et al.*, 2004). A three-component condensation of urea derivatives with aromatic aldehydes and β-ketoesters, known as Biginelli reaction allows the synthesis of the DHPM unit with a widely modified and variable substitution pattern. It was proposed that the combination of the biologically active DHPM subunit with a DNA intercalator (Waring, 2006; Hannon, 2007; Ihmels & Otto, 2005) may lead to a new class of DNA-targeting drugs (Neidle & Thurston, 2005; Braña *et al.*, 2001). Thus, a classical DNA, namely quinoline (Denny, 2003; Kharatishvili *et al.*, 1997; Aislabie *et al.*, 1990), was employed as quinoline-4-carbaldehyde in the Biginelli reaction according to traditional reaction conditions in ethanol employing hydrochloric acid as catalyst (Biginelli, 1893) leading to the title compound (Fig. 1).

Tetrahydropyrimidone ring adopts a distorted boat conformation with the C1, C2, C3 and N1 atoms almost coplanar (dihedral angle of 1.47 (18)°), while the C4 and N2 atoms deviated from this plane by -0.342 (2) Å and -0.481 (2) Å, respectively. This non-planariry is probably additionally supported by steric repulsion between methyl groups at the C3 and N1 atoms (short intramolecular contacts C17···H18*a* 2.55 Å and C18···C17*a* 2.57 Å while van der Waals radii sum is 2.87 Å, Zefirov & Zorky, 1989). Quinoline substituent has axial orientation (the C4—N2—C1—C5 torsion angle of -95.03 (14)°) and rotated almost coplanar to the C1—C2 bond (dihedral angle of -13.55 (16)°) despite short intramolecular contact H6···C2 2.48 Å (van der Waals radii sum is 2.87 Å, Zefirov & Zorky, 1989). Ester substituent at the C2 atom slightly rotated with respect to C1/C2/C3/N1 fragment (the C1—C2—C14—O1 torsion angle of -24.96 (18)°) supporting formation of attractive intramolecular contact O1···H1 2.44 Å (van der Waals radii sum of 2.46 Å). The ester substituent rotation is accompanied by formation of short intramolecular C17—H17c···O2 hydrogen bond.

The crystal packing (Fig. 2) is stabilized by intramolecular C—H···O and intermolecular N—H···O hydrogen bonds between adjacent amide fragments of pyrimidine ring, with a N2—H2···O3^i^ (Table 1). The molecular packing (Fig. 2) is further stabilized by an intermolecular C—H···π interaction between the methyl H atom of the ethyl group and the pyridine ring of a neighbouring quinoline system, with a C16—H16C···Cg1^ii^.

(Table 1; Cg1 is the centroid of the C5/C6/C7/N3/C8/C13 pyridine ring).

### Experimental

The title compound was synthesized by refluxing condition of monomethylurea (0.237 g, 3.20 mmol), 4-quinoline aldehyde (0.502 g, 3.20 mmol), acetoacetic ester (0.520 g, 4.00 mmol) with catalytic amount of HCl in 15 ml EtOH. The residue was purified by column chromatography (silica gel, hexane–acetone, 90:5 v/v) to afforded the title compound as a colorless solid (yield 48%, m.p. 472 K). The single crystals suitable for X-ray diffraction were obtained by evaporation of a solution of the title compound in acetone.

### Refinement

All H-atoms were positioned geometrically and refined using a riding model with d (N—H) = 0.86 Å, d (C—H) = 0.93–0.98 Å and *U*_iso_(H) = 1.2–1.5 *U*_eq_ (parent atom).

### Crystal data

**Table:**

C_18_H_19_N_3_O_3_	*Z* = 2
*M**_r_* = 325.36	*F*(000) = 344
Triclinic, *P*1	*D*_x_ = 1.364 Mg m^−^^3^
*a* = 8.5039 (10) Å	Mo *K*α radiation, λ = 0.7107 Å
*b* = 9.4637 (11) Å	Cell parameters from 3300 reflections
*c* = 10.7503 (11) Å	θ = 3.1–32.4°
α = 110.799 (10)°	µ = 0.10 mm^−^^1^
β = 95.807 (9)°	*T* = 293 K
γ = 97.339 (9)°	Prism, colorless
*V* = 792.07 (17) Å^3^	0.4 × 0.3 × 0.2 mm

### Data collection

**Table:**

Oxford Diffraction Xcalibur diffractometer with Sapphire3 detector	4607 independent reflections
graphite	2810 reflections with *I* > 2σ(*I*)
Detector resolution: 16.1827 pixels mm^-1^	*R*_int_ = 0.017
ω scans	θ_max_ = 30.0°, θ_min_ = 3.1°
Absorption correction: multi-scan (*CrysAlis PRO*; Oxford Diffraction, 2009)	*h* = −12→12
*T*_min_ = 0.975, *T*_max_ = 1	*k* = −14→13
8517 measured reflections	*l* = −14→15

### Refinement

**Table:**

Refinement on *F*^2^	Primary atom site location: structure-invariant direct methods
Least-squares matrix: full	Secondary atom site location: difference Fourier map
*R*[*F*^2^ > 2σ(*F*^2^)] = 0.044	Hydrogen site location: inferred from neighbouring sites
*wR*(*F*^2^) = 0.117	H-atom parameters constrained
*S* = 0.99	*w* = 1/[σ^2^(*F*_o_^2^) + (0.060*P*)^2^] where *P* = (*F*_o_^2^ + 2*F*_c_^2^)/3
4607 reflections	(Δ/σ)_max_ = 0.001
220 parameters	Δρ_max_ = 0.30 e Å^−^^3^
0 restraints	Δρ_min_ = −0.17 e Å^−^^3^

### Special details

**Table:**

Geometry. All esds (except the esd in the dihedral angle between two l.s. planes) are estimated using the full covariance matrix. The cell esds are taken into account individually in the estimation of esds in distances, angles and torsion angles; correlations between esds in cell parameters are only used when they are defined by crystal symmetry. An approximate (isotropic) treatment of cell esds is used for estimating esds involving l.s. planes.
Refinement. Refinement of *F*^2^ against ALL reflections. The weighted *R*-factor wR and goodness of fit *S* are based on *F*^2^, conventional *R*-factors *R* are based on *F*, with *F* set to zero for negative *F*^2^. The threshold expression of *F*^2^ > σ(*F*^2^) is used only for calculating *R*-factors(gt) etc. and is not relevant to the choice of reflections for refinement. *R*-factors based on *F*^2^ are statistically about twice as large as those based on *F*, and *R*- factors based on ALL data will be even larger.

### Fractional atomic coordinates and isotropic or equivalent isotropic displacement parameters (Å^2^)

**Table:**

	*x*	*y*	*z*	*U*_iso_*/*U*_eq_	
O1	0.27163 (12)	0.04049 (12)	0.18120 (11)	0.0531 (3)	
O2	0.06327 (12)	−0.03288 (11)	0.26749 (11)	0.0499 (3)	
O3	−0.17806 (12)	0.43713 (11)	0.03648 (11)	0.0505 (3)	
N1	−0.19236 (12)	0.24916 (11)	0.12189 (11)	0.0342 (2)	
N2	0.05174 (13)	0.36177 (12)	0.09001 (11)	0.0392 (3)	
H2	0.1019	0.4088	0.0464	0.047*	
N3	0.34211 (15)	0.63743 (12)	0.56698 (12)	0.0471 (3)	
C1	0.14609 (14)	0.29466 (13)	0.16757 (12)	0.0315 (3)	
H1	0.2345	0.2599	0.1197	0.038*	
C2	0.04233 (15)	0.15552 (12)	0.17321 (12)	0.0317 (3)	
C3	−0.11880 (15)	0.13846 (13)	0.15232 (12)	0.0324 (3)	
C4	−0.10723 (16)	0.35587 (13)	0.08131 (13)	0.0355 (3)	
C5	0.21887 (13)	0.41308 (12)	0.30752 (12)	0.0297 (3)	
C6	0.16516 (16)	0.40622 (14)	0.42047 (13)	0.0377 (3)	
H6	0.0850	0.3261	0.4139	0.045*	
C7	0.23041 (18)	0.51977 (16)	0.54688 (15)	0.0467 (3)	
H7	0.1913	0.5106	0.6219	0.056*	
C8	0.40063 (15)	0.64740 (14)	0.45630 (14)	0.0385 (3)	
C9	0.52172 (17)	0.77391 (15)	0.47649 (16)	0.0497 (4)	
H9	0.5580	0.8462	0.5631	0.060*	
C10	0.58558 (18)	0.79118 (17)	0.37115 (18)	0.0562 (4)	
H10	0.6645	0.8754	0.3856	0.067*	
C11	0.53288 (17)	0.68249 (17)	0.24086 (17)	0.0519 (4)	
H11	0.5785	0.6942	0.1693	0.062*	
C12	0.41510 (15)	0.55914 (15)	0.21731 (15)	0.0390 (3)	
H12	0.3810	0.4886	0.1298	0.047*	
C13	0.34461 (13)	0.53745 (13)	0.32394 (13)	0.0315 (3)	
C14	0.13704 (16)	0.04890 (13)	0.20431 (13)	0.0356 (3)	
C15	0.14436 (18)	−0.14639 (16)	0.29605 (17)	0.0488 (4)	
H15B	0.2456	−0.0972	0.3555	0.059*	
H15A	0.1659	−0.2207	0.2133	0.059*	
C16	0.0369 (2)	−0.2227 (2)	0.3611 (2)	0.0799 (7)	
H16B	0.0163	−0.1479	0.4426	0.120*	
H16C	0.0869	−0.2987	0.3820	0.120*	
H16A	−0.0625	−0.2712	0.3011	0.120*	
C17	−0.23434 (17)	0.00572 (15)	0.15444 (17)	0.0485 (4)	
H17C	−0.2872	0.0395	0.2319	0.073*	
H17A	−0.1769	−0.0738	0.1587	0.073*	
H17B	−0.3128	−0.0334	0.0741	0.073*	
C18	−0.36752 (16)	0.23559 (17)	0.09982 (16)	0.0475 (4)	
H18B	−0.4112	0.2070	0.1682	0.071*	
H18A	−0.4111	0.1585	0.0127	0.071*	
H18C	−0.3949	0.3324	0.1041	0.071*	

### Atomic displacement parameters (Å^2^)

**Table:**

	*U*^11^	*U*^22^	*U*^33^	*U*^12^	*U*^13^	*U*^23^
O1	0.0463 (6)	0.0626 (6)	0.0668 (8)	0.0204 (5)	0.0161 (5)	0.0384 (6)
O2	0.0498 (6)	0.0499 (6)	0.0725 (8)	0.0210 (4)	0.0171 (5)	0.0433 (5)
O3	0.0463 (6)	0.0575 (6)	0.0623 (7)	0.0094 (4)	0.0002 (5)	0.0421 (5)
N1	0.0347 (6)	0.0355 (5)	0.0355 (6)	0.0048 (4)	0.0016 (5)	0.0183 (4)
N2	0.0379 (6)	0.0459 (6)	0.0416 (7)	0.0001 (4)	−0.0010 (5)	0.0301 (5)
N3	0.0521 (7)	0.0459 (6)	0.0369 (7)	0.0003 (5)	0.0004 (5)	0.0124 (5)
C1	0.0329 (6)	0.0319 (6)	0.0324 (7)	0.0051 (4)	0.0036 (5)	0.0158 (5)
C2	0.0381 (7)	0.0275 (5)	0.0289 (6)	0.0034 (4)	0.0012 (5)	0.0116 (5)
C3	0.0402 (7)	0.0296 (6)	0.0269 (6)	0.0027 (5)	0.0009 (5)	0.0121 (5)
C4	0.0411 (7)	0.0368 (6)	0.0296 (7)	0.0030 (5)	−0.0012 (5)	0.0167 (5)
C5	0.0295 (6)	0.0295 (5)	0.0328 (7)	0.0078 (4)	0.0026 (5)	0.0146 (5)
C6	0.0416 (7)	0.0358 (6)	0.0359 (7)	0.0016 (5)	0.0053 (6)	0.0157 (5)
C7	0.0571 (9)	0.0482 (8)	0.0337 (8)	0.0036 (6)	0.0072 (7)	0.0159 (6)
C8	0.0363 (7)	0.0372 (7)	0.0396 (8)	0.0042 (5)	−0.0017 (6)	0.0144 (6)
C9	0.0433 (8)	0.0428 (8)	0.0538 (10)	−0.0051 (6)	−0.0066 (7)	0.0151 (6)
C10	0.0433 (9)	0.0512 (9)	0.0716 (12)	−0.0093 (6)	−0.0034 (8)	0.0291 (8)
C11	0.0426 (8)	0.0599 (9)	0.0618 (11)	0.0003 (7)	0.0095 (7)	0.0354 (8)
C12	0.0341 (7)	0.0441 (7)	0.0423 (8)	0.0059 (5)	0.0042 (6)	0.0210 (6)
C13	0.0271 (6)	0.0326 (6)	0.0380 (7)	0.0067 (4)	0.0012 (5)	0.0174 (5)
C14	0.0416 (7)	0.0309 (6)	0.0335 (7)	0.0057 (5)	0.0023 (6)	0.0123 (5)
C15	0.0510 (8)	0.0454 (8)	0.0646 (10)	0.0194 (6)	0.0091 (7)	0.0341 (7)
C16	0.0721 (12)	0.0777 (12)	0.136 (2)	0.0347 (10)	0.0407 (13)	0.0815 (13)
C17	0.0440 (8)	0.0434 (7)	0.0608 (10)	−0.0029 (6)	−0.0011 (7)	0.0289 (7)
C18	0.0372 (8)	0.0574 (8)	0.0553 (10)	0.0096 (6)	0.0063 (7)	0.0297 (7)

### Geometric parameters (Å, °)

**Table:**

O1—C14	1.2018 (15)	C8—C9	1.4125 (18)
O2—C14	1.3377 (15)	C8—C13	1.4207 (18)
O2—C15	1.4504 (14)	C9—C10	1.356 (2)
O3—C4	1.2283 (14)	C9—H9	0.9300
N1—C4	1.3871 (15)	C10—C11	1.397 (2)
N1—C3	1.4019 (14)	C10—H10	0.9300
N1—C18	1.4662 (16)	C11—C12	1.3676 (18)
N2—C4	1.3380 (16)	C11—H11	0.9300
N2—C1	1.4538 (14)	C12—C13	1.4103 (18)
N2—H2	0.8600	C12—H12	0.9300
N3—C7	1.3061 (17)	C15—C16	1.471 (2)
N3—C8	1.3637 (18)	C15—H15B	0.9700
C1—C2	1.5122 (15)	C15—H15A	0.9700
C1—C5	1.5293 (17)	C16—H16B	0.9600
C1—H1	0.9800	C16—H16C	0.9600
C2—C3	1.3451 (17)	C16—H16A	0.9600
C2—C14	1.4735 (16)	C17—H17C	0.9600
C3—C17	1.5012 (16)	C17—H17A	0.9600
C5—C6	1.3591 (17)	C17—H17B	0.9600
C5—C13	1.4330 (15)	C18—H18B	0.9600
C6—C7	1.4022 (19)	C18—H18A	0.9600
C6—H6	0.9300	C18—H18C	0.9600
C7—H7	0.9300		
			
C14—O2—C15	117.81 (10)	C9—C10—H10	120.0
C4—N1—C3	121.22 (10)	C11—C10—H10	120.0
C4—N1—C18	115.73 (10)	C12—C11—C10	120.81 (14)
C3—N1—C18	121.48 (10)	C12—C11—H11	119.6
C4—N2—C1	124.96 (10)	C10—C11—H11	119.6
C4—N2—H2	117.5	C11—C12—C13	120.95 (13)
C1—N2—H2	117.5	C11—C12—H12	119.5
C7—N3—C8	116.88 (12)	C13—C12—H12	119.5
N2—C1—C2	108.97 (10)	C12—C13—C8	117.82 (11)
N2—C1—C5	111.30 (9)	C12—C13—C5	124.39 (12)
C2—C1—C5	112.65 (10)	C8—C13—C5	117.79 (11)
N2—C1—H1	107.9	O1—C14—O2	122.41 (11)
C2—C1—H1	107.9	O1—C14—C2	123.48 (11)
C5—C1—H1	107.9	O2—C14—C2	114.03 (11)
C3—C2—C14	126.27 (11)	O2—C15—C16	106.99 (11)
C3—C2—C1	120.96 (10)	O2—C15—H15B	110.3
C14—C2—C1	112.77 (10)	C16—C15—H15B	110.3
C2—C3—N1	119.79 (10)	O2—C15—H15A	110.3
C2—C3—C17	125.97 (11)	C16—C15—H15A	110.3
N1—C3—C17	114.21 (11)	H15B—C15—H15A	108.6
O3—C4—N2	122.75 (11)	C15—C16—H16B	109.5
O3—C4—N1	120.25 (12)	C15—C16—H16C	109.5
N2—C4—N1	116.98 (10)	H16B—C16—H16C	109.5
C6—C5—C13	117.48 (11)	C15—C16—H16A	109.5
C6—C5—C1	121.55 (10)	H16B—C16—H16A	109.5
C13—C5—C1	120.96 (10)	H16C—C16—H16A	109.5
C5—C6—C7	120.17 (12)	C3—C17—H17C	109.5
C5—C6—H6	119.9	C3—C17—H17A	109.5
C7—C6—H6	119.9	H17C—C17—H17A	109.5
N3—C7—C6	124.77 (13)	C3—C17—H17B	109.5
N3—C7—H7	117.6	H17C—C17—H17B	109.5
C6—C7—H7	117.6	H17A—C17—H17B	109.5
N3—C8—C9	117.53 (13)	N1—C18—H18B	109.5
N3—C8—C13	122.88 (11)	N1—C18—H18A	109.5
C9—C8—C13	119.59 (13)	H18B—C18—H18A	109.5
C10—C9—C8	120.78 (14)	N1—C18—H18C	109.5
C10—C9—H9	119.6	H18B—C18—H18C	109.5
C8—C9—H9	119.6	H18A—C18—H18C	109.5
C9—C10—C11	120.05 (13)		
			
C4—N2—C1—C2	29.78 (16)	C8—N3—C7—C6	−1.4 (2)
C4—N2—C1—C5	−95.05 (13)	C5—C6—C7—N3	0.6 (2)
N2—C1—C2—C3	−21.57 (16)	C7—N3—C8—C9	−179.87 (13)
C5—C1—C2—C3	102.47 (13)	C7—N3—C8—C13	0.41 (19)
N2—C1—C2—C14	159.04 (10)	N3—C8—C9—C10	179.93 (13)
C5—C1—C2—C14	−76.92 (13)	C13—C8—C9—C10	−0.3 (2)
C14—C2—C3—N1	−179.22 (11)	C8—C9—C10—C11	−0.6 (2)
C1—C2—C3—N1	1.47 (18)	C9—C10—C11—C12	1.0 (2)
C14—C2—C3—C17	−1.0 (2)	C10—C11—C12—C13	−0.6 (2)
C1—C2—C3—C17	179.65 (12)	C11—C12—C13—C8	−0.31 (18)
C4—N1—C3—C2	15.51 (18)	C11—C12—C13—C5	178.85 (12)
C18—N1—C3—C2	−179.37 (12)	N3—C8—C13—C12	−179.52 (11)
C4—N1—C3—C17	−162.87 (11)	C9—C8—C13—C12	0.77 (17)
C18—N1—C3—C17	2.25 (17)	N3—C8—C13—C5	1.27 (17)
C1—N2—C4—O3	165.93 (12)	C9—C8—C13—C5	−178.45 (11)
C1—N2—C4—N1	−15.95 (18)	C6—C5—C13—C12	178.85 (11)
C3—N1—C4—O3	169.42 (12)	C1—C5—C13—C12	−2.32 (17)
C18—N1—C4—O3	3.50 (18)	C6—C5—C13—C8	−1.99 (15)
C3—N1—C4—N2	−8.75 (18)	C1—C5—C13—C8	176.84 (10)
C18—N1—C4—N2	−174.68 (12)	C15—O2—C14—O1	−6.3 (2)
N2—C1—C5—C6	109.13 (12)	C15—O2—C14—C2	176.97 (11)
C2—C1—C5—C6	−13.61 (15)	C3—C2—C14—O1	155.76 (13)
N2—C1—C5—C13	−69.65 (13)	C1—C2—C14—O1	−24.89 (18)
C2—C1—C5—C13	167.60 (10)	C3—C2—C14—O2	−27.58 (19)
C13—C5—C6—C7	1.16 (17)	C1—C2—C14—O2	151.78 (11)
C1—C5—C6—C7	−177.67 (11)	C14—O2—C15—C16	−178.22 (14)

### Hydrogen-bond geometry (Å, °)

**Table:**

Cg1 is the centroid of the C8–C13 ring.

**Table:**

*D*—H···*A*	*D*—H	H···*A*	*D*···*A*	*D*—H···*A*
N2—H2···O3^i^	0.86	2.03	2.8701 (16)	164
C17—H17A···O2	0.96	2.16	2.8187 (19)	124
C16—H16C···Cg1^ii^	0.96	2.66	3.603 (2)	168

Symmetry codes: (i) −*x*, −*y*+1, −*z*; (ii) *x*, *y*−1, *z*.
